# Evaluating State-of-the-Art Computerized Pupillary Assessments for Glaucoma Detection: A Systematic Review and Meta-Analysis

**DOI:** 10.3389/fneur.2020.00777

**Published:** 2020-07-29

**Authors:** Lingge Suo, Di Zhang, Xuejiao Qin, Annan Li, Chun Zhang, Yunhong Wang

**Affiliations:** ^1^Beijing Key Laboratory of Restoration of Damaged Ocular Nerve, Department of Ophthalmology, Peking University Third Hospital, Beijing, China; ^2^Department of Ophthalmology, Qilu Hospital of Shandong University, Jinan, China; ^3^Laboratory of Intelligent Recognition and Image Processing, School of Computer Science and Engineering, Beihang University, Beijing, China

**Keywords:** glaucoma, pupil light reflex (PLR), automated pupillography, chromatic pupillometry, ipRGC, meta-analysis

## Abstract

Computerized pupillary light reflex assessment devices (CPLRADs) may serve as an effective screening tool for glaucomatous optic neuropathy, since they can dynamically detect abnormal pupillary responses from a novel sequence of light stimuli and functionally-shaped stimuli. The aim of this study was to systematically evaluate the current state of advanced CPLRADs and accuracy of application in detecting glaucoma. An electronic literature search of PubMed, MEDLINE, and Embase from database inception to December 2019 was performed. Studies that reported data on the use of computer-aided pupillometry with monocular and/or binocular monitoring in glaucoma patients were included. Two review authors independently conducted the study selection and extracted study data. A total of twenty-five studies were included in this review; eight studies with a total of 829 subjects were included in this meta-analysis. Data were pooled using a random-effect model, since the significant heterogeneity (*P* < 0.1, *I*^2^> 50%). Our meta-analysis of eight studies showed reasonably high summary sensitivity and specificity estimates of 0.81 (95% CI 0.73–0.89) and 0.83 (95% CI: 0.75–0.91), respectively. Simpler monochromatic devices, such as Pupilmetrix^TM^ PLR60, generally performed as well as or slightly better than more complex chromatic devices. This review suggests that CPLRADs may facilitate direct clinical decision making for glaucoma diagnosis and evaluation, and may provide a deeper understanding of the pathomechanism of glaucoma.

## 1. Introduction

Glaucoma comprises a heterogeneous group of diseases characterized by progressive loss of retinal ganglion cells (RGCs) and their optic nerve axons, discernable by cupping of the optic nerve head, with associated visual-field damage or even blindness (1). Since glaucomatous visual field loss is irreversible, early detection, diagnosis and treatment are important for glaucoma screening. It is estimated that the number of people with glaucoma will be more than 110 million by globally 2040 (2). All over the world, detection and mass screening of glaucoma remains a challenge (3). Thus, advanced automated, inexpensive and non-invasive screen tools are vital to prevent glaucoma from progressing to advanced stages and to minimize future healthcare costs and thereby ease the globe eye healthcare burden.

The pupillary light reflex (PLR) is driven by photoreceptors [i.e., rods, cones, and intrinsically photosensitive retinal ganglion cells (ipRGCs)] that control pupil dilation or constriction in response to light that falls on the retina. Consequently, how much light enters the eye can be modified, aiding the adaptation to various levels of darkness and light (4). During ophthalmological examination, this physiological reaction is routinely elicited as a functional marker of the retina, the optic nerve, and even the brain stem (5). PLR abnormalities often manifest as a relative afferent pupillary defect (RAPD) and based on a variety of conditions that involve the integrity of the entire visual pathway (6). In glaucoma, asymmetric damage is usually located between the upper and lower retina and the loss of retinal nerve fibers is often more severe in one eye. A previous review assessing the PLR found that patients with glaucoma often had abnormal RAPD or PLR (5). Therefore, the PLR as a window to glaucoma screening, objectively measures the pupil dynamic parameters monocularly and/or binocularly as indicators from the retina and optic nerve (7).

In routine clinics, although visual field (VF) testing by standard automated perimetry (SAP) is efficient in detecting functional changes, the test remains subjective and time-consuming (8, 9). Additionally, the optical coherence tomography (OCT) is efficient at detecting structural changes in glaucoma, but this approach is susceptible to the effect of outliers, and the results can be inappropriately labeled as progression; therefore, there is a need for multiple tests before the results can be considered reliable (10). Furthermore, in the clinic, the swinging flashlight test (SFT), conducted with or without using neutral density filters to evaluate RAPD is a manual and subjective qualitative test. It is not easy to master the various sources of erroneous results (11, 12). Thus, VF, OCT, and SFT currently lack the necessary diagnostic performance for glaucoma screening of the general population.

**Figure 1:**
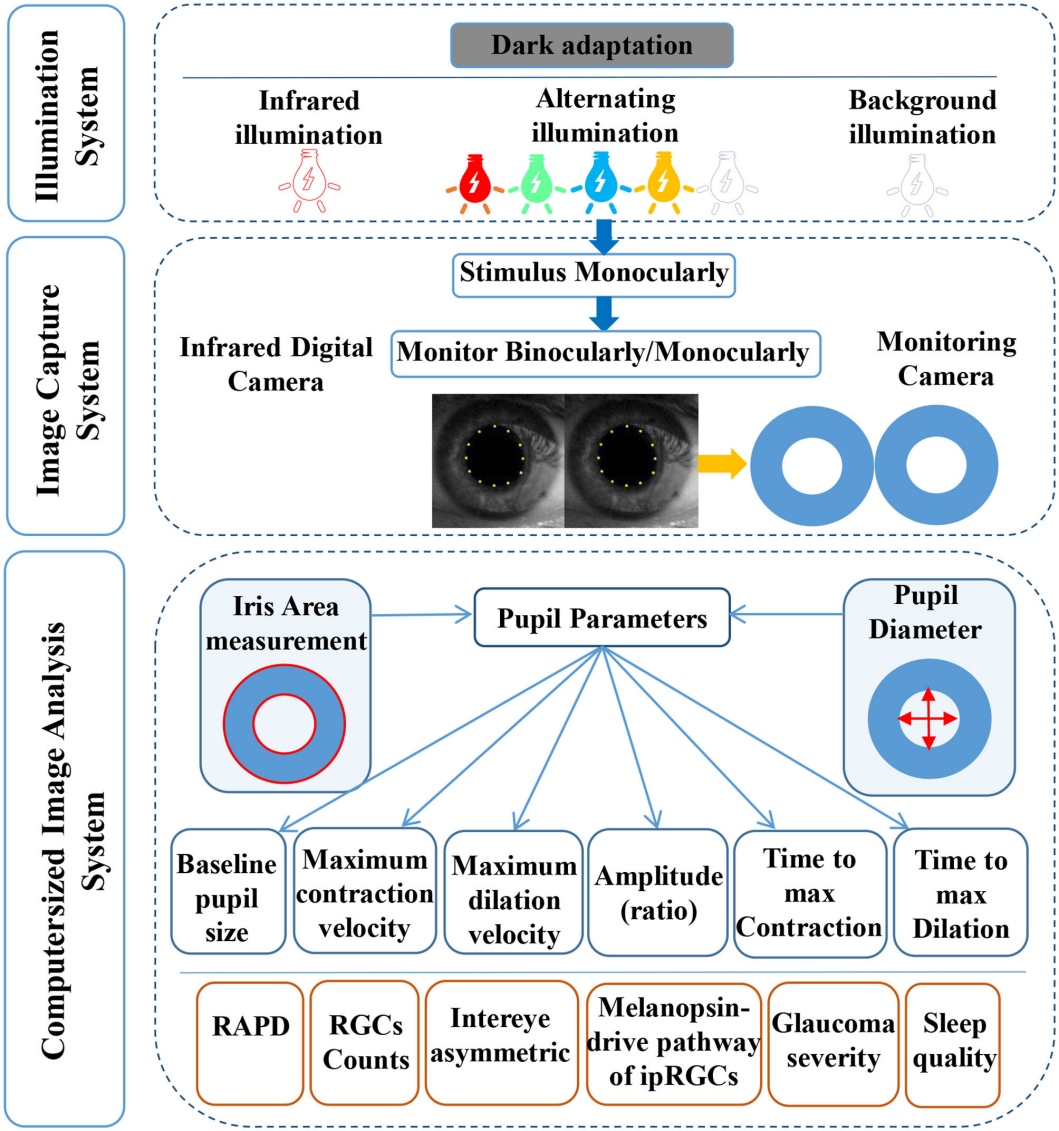
A diagram of the computerized pupillary light reflex assessment devices. CPLRADs contain three main parts: an illumination system (infrared illumination, alternating color illumination, background illumination, functionally-shaped stimuli program), an image capturing system (infrared digital camera, monitoring camera), and a computerized image analysis system (capturing the following pupil parameters: baseline pupillary size, maximum contraction velocity, maximum dilation velocity, amplitude (ratio), time to max contraction, time to maximal dilation).

CPLRADs are able to dynamically measure the entire waveform of the pupillary response under a novel sequence of controlled color light stimuli and functionally-shaped stimuli, possibly detecting and evaluating glaucomatous optic neuropathy (13–16) (Figure 1). These devices record the amplitudes, latencies and velocities of the PLR to present unilateral and bilateral metrics, predicting the between-two-eyes and within-one-eye asymmetry in the structural and functional damage to the retina and optic nerve (17, 18). To our knowledge, chromatic pupillometry as a tool can evaluate the integrity of visual functions and localize retinal dysfunctions in different ocular diseases, especially glaucoma. PLRs are based on the functionality of photoreceptors within the inner (red/green-shifted peak sensitivity) and outer (blue-shifted peak sensitivity) retina, as well as the integrity of their neural circuitry (19). Evidence of the involvement of ipRGCs in the PLRs under novel color-variant and functionally-shaped CPLRAD methods has also been accumulating.

In this study, we reviewed state-of-the-art CPLRAD techniques for measuring and quantifying the PLRs in glaucoma patients. Furthermore, since the stimulus presented can be varied in color, shape, intensity, duration, and size, we assessed the accuracy of using those techniques for direct clinical decision making for glaucoma diagnosis and evaluation.

## 2. Materials and Methods

We performed a systematic review according to the Preferred Reporting Items for Systematic Reviews and Meta-analysis (PRISMA) statement. The protocol was developed at the start of our investigation. Ethical approval and patient consent were not required, because all data analyses were performed with previously published studies.

### 2.1. Data Sources and Search Strategy

Systematic literature research using the PubMed, MEDLINE, and Embase databases, as well as references of the included studies, was performed from database inception through a search end date of December 10, 2019. For PubMed, Embase, and MEDLINE, we used both controlled vocabulary and text words for synonymous terminology within titles and abstracts in the development of search strategies. The search strategy contained all possible combinations of terms: chromatic pupillometry, automated pupillography, pupillometer, pupillary light reflex, PLR, relative afferent pupillary defect, RAPD, pupil, glaucoma, visual field defect, retinal nerve fiber layer thickness, RNFL thickness, retinal ganglion cell loss (Table S1). Before the final analysis, two review authors reran our search protocol to confirm the results and searched Google Scholar and Web of Science, to identify additional studies.

### 2.2. Inclusion and Exclusion Criteria

Articles reporting the use of CPLRADs to examine abnormal PLRs/RAPDs in glaucoma were eligible if they met all of the following criteria: (1) recruited participants with diagnosed glaucomatous optic neuropathy; (2) used samples of participants aged 18 years or over; (3) utilized an acceptable reference standard; and (4) reported sufficient results to calculate metrics for the diagnostic accuracy of the technique used.

An article was excluded if: (1) testing was performed on infants/toddlers; (2) the number of participants with glaucoma was <10; or (3) the study was not published in English or was a review study, letter, or conference paper.

### 2.3. Data Collection

Two reviewers independently extracted the data of selected articles using a piloted data extraction form. Disagreements between individual judgments were resolved by discussions with a senior reviewer (XJQ). The extracted information included the title, authors, publication year, study design, characteristics of each study population (including the sample size and mean age), equipment for measuring pupillary responses, binocular or monocular study category, stimulus color, stimulus pattern, and main outcomes (Table 1, Table S2). The aim of our review was to evaluate the application of computer-aided pupillary assessment technology to glaucoma diagnosis and evaluation. Therefore, we assessed the ability and accuracy of the pupillary response measuring tool in detecting glaucoma. The accuracy was quantified by the sensitivity and specificity or AUC of each study.

**Table 1:** The characteristics of studies included in the meta-analysis.

**Source**	**Study**	**Age, year**	**Device**	**B/M**	**DAT**	**Stimuli**	**S S**	**Structural**	**Main**
	**design**		**name**			**C**	**Pattern**	**functional**	**results**
								**and**	
								**asymmetry**	
Pillai et al. (16)	Case-control	G56.9	RAPiDo	B	U	c	U	Significant	Sensitivity: 89%
India	study	H35.21						MD, CDR asy	Specificity: 91.7%
	G130, H43							(*p* < 0.001)	AUC: 0.94
	Prospective							Median MD	Amp-FF-W: Sensitivity:
Rao et al. (20)	Cross-sectional	G61	RAPDx	B	1–2 min	w/b/g/r/y	Peripheral/Full	asy = 6.7 dB	5–45%;
India	study	H46					field	(range: 0.1–27.2	Specificity: 95%;
	G47, H42							dB)	AUC: 0.60–0.82
	Cross-sectional								Standard setting: Sensitivity:
Waisbourd et	study	66.2 ± 13.6	RAPDx	B	1–2 min	w	Full-field	MD asy > 5 dB;	93.3%;Specificity:
al. (21), USA	G60							CDR asy≥0.20	41.2%; AUC: 0.84 for
	OH/GS21								detecting MD asymmetry
	Cross-sectional							Mean MD asy:	Full field (ConMaxVelLatR)
Tatham et al. (7)	study	G69.1	RAPDx	B/M	U	w/b/g/r/y	Peripheral/Full	2.2 ± 3.1dB;	Sensitivity: 53%;
USA	G66, H50	H51.3					field	Significant MD	Specificity: 80%; AUC: 0.75
	Prospective							asy (*p* < 0.001)	(green stimulus)
									Full model:
Chang et al. (5)	Case-control	G67 ± 11	RAPDx	B/M	1 min	w/b/g/r/y	Peripheral/Full	MD asy < −5 dB;	Sensitivity 84%;
USA	study	H60 ± 10					field		Specificity 80%;
	G148, H71								AUROC: 0.90
Kalaboukhova	Case-control	G65 ± 10	A custom-	B	15 s	w	Peripheral/Full	Mean MD:	PARm (a cut-off point of
et al. (22)	study	H63 ± 8	built				field	6.3 dB	1.16) Sensitivity
Sweden	G30, H30		pupillometer					(range: 0.31–	86.7%; Specificity
								18.80 dB)	90%; AUROC 0.923
	Case-control			A			Paracentral/		Peripheral stimulus pattern:
Wride et al. (23)	study	G59.6 ± 16.7	Pupilmetrix^TM^	M	short	w	Bjerrum/	U	Sensitivity 93.1%;
UK	G29, H30	H69.9 ± 13.6	PLR60		period		Peripheral		Specificity 76.7%;
									AUROC 0.907
Kalaboukhova	Case-control	G69 ± 7	A custom-	B	15 s	w	Peripheral/Full	Minimum MD	PARm (a cut-off point
et al. (24)	study	H59 ± 14	built				field	asy: 1.6 dB	of 1.16): Sensitivity
Sweden	G17, H15		pupillometer						82.4; Specificity
									86.7; AUROC 0.929

*Y, year; B/M, Binocular/Monocular monitoring; DAT, Dark adaptation time; Stimuli C, stimuli color; S S Pattern, stimuli shape pattern; U, unclear; w, white; b, blue; g, green; r, red; y, yellow; AUROC, area under the receiver operating characteristics curve; G, glaucoma patients; H, health control; OH, ocular hypertension; GS, glaucoma suspect; asy, asymmetry; MD, Mean Deviation; CDR, cup to disc ratio; ConMaxVelLat, latency of the maximum velocity of constriction, PARm, mean value of pupillary area ratio for three measurements; U, unclear*.

### 2.4. Risk of Bias/Quality Assessment

Each of the full text articles was independently reviewed by two reviewers (LGS, DZ) and scored with the Quality Assessment of Diagnostic Accuracy Studies 2 scores (QUADAS-2) tool. The methodologic quality of the included studies was evaluated with the QUADAS-2 tool. The risk of bias was assessed in four domains (i.e., patient selection, index test, reference standard, and flow and timing). The applicability concerns were assessed in three domains (i.e., patient selection, index test, reference standard). Each domain was assessed by indicating a “low,” “high,” or “unclear” rating.

### 2.5. Statistical Analysis

We used Stata software version 15.0 (StataCorp, College Station, TX) to assess meta analysis. Extracted data were synthesized by creating forest plots of sensitivity and specificity. Heterogeneity was evaluated by Cochrane's *Q*-test (*I*^2^ value); heterogeneity was considered to exist when the *p*-value was > 0.1. When *I*^2^ results were ≤ 50%, a fixed effects model was used; otherwise, a random effect model was used. Sensitivity analysis was used to evaluate the stability of the results. The above results were considered statistically significant when the *p*-value was < 0.05. We further explored our results using preplanned subgroup analyses that included only studies without missing information with respect to chromatic light stimulus or monochromatic light stimulus analysis.

## 3. Results

### 3.1. Search Results

**Figure 2:**
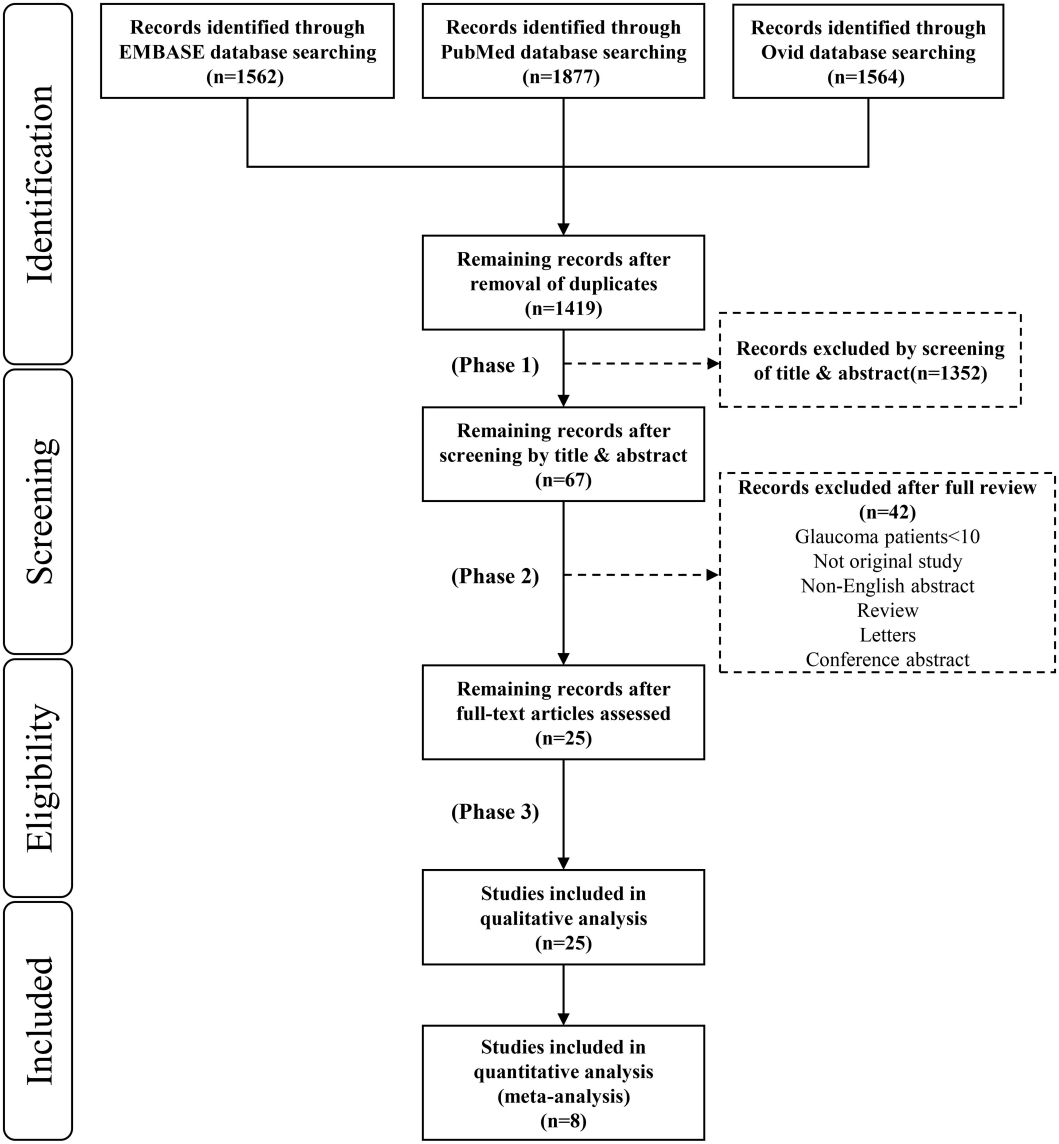
Preferred reporting items for systematic reviews and meta-analyses (PRISMA) flow diagram for study inclusion.

The initial literature search identified 5,003 citations (1,562 articles in Embase, 1,877 articles in PubMed and 1,564 articles in Medline). Figure 2 shows the process of selecting the 25 studies included in our analysis. After we removed duplicates from the three databases 1,419 records remained. In the first phase, we screened each record based on its title and abstract and 1,352 studies with no reference to the pupil and glaucoma were excluded. In the second phase, we excluded studies that were non-English, reviews, letters or conference abstracts; studies with glaucoma patients <10 were also excluded. In the third phase, we enrolled 25 studies in the quality assessment. Finally, we evaluated eight studies in the quantitative analysis (Table 1).

### 3.2. Study Characteristics

Table 1 reports which CPLRAD test was used in each study investigated, as well as the characteristics of the study participants, stimulus color, stimulus shape pattern, reference standard utilized and diagnostic accuracy in the meta-analysis. Four types of devices, based on color-variant stimuli, were evaluated and divided into two groups: a monochromatic light group and a chromatic light group. Seven studies presented bilateral metrics by monitoring RAPDs in glaucoma, predicting the between-two-eyes asymmetry in structural and functional damage to the retina and optic nerve (7, 16, 20–22, 24, 25). The main results showed the sensitivity and specificity of each method in relation to inter-eye differences in mean deviation (MD). Three studies presented unilateral metrics by monitoring the PLR in different parts of the visual field (e.g., asymmetry between the superior and inferior visual fields) within the same eye, predicting the within-one-eye asymmetry in structural and functional damage to the retina and optic nerve in glaucoma (7, 23, 25). For each variable, the CPLRAD tests had high sensitivities for clinically significant cut points.

Table S2 reports the clinical data of the enrolled participants in studies using one of nine types of devices. Thirteen studies evaluated correlations between CPLRAD parameters and ophthalmic features [e.g., MD, retinal nerve fiber layer (RNFL) thickness, and ganglion cell complex (GCC) thickness] in glaucoma (14, 15, 18, 26–35). Eight studies investigated the PLR driven by ipRGCs and classical photoreceptors and found the inner and outer retinal contributions in glaucoma (14, 17, 27, 32, 34–37).

In summary of Table 1 and Table S2, the included studies were conducted in 10 countries and were published in English. Nine (36%) of 25 studies were performed in the USA, 5 (20%) in India, 2 (8%) in Sweden, 1 (4%) in UK, 2 (8%) in Singapore, 1 (4%) in Germany, 1 (4%) in Italy, 3 (12%) in Australia, 1 (4%) in Denmark, and 1 (4%) in Japan. The sample size ranged from 22 to 219. In each study, both men and women were recruited. The average age of the glaucoma participants ranged from 49 to 78 years; the average age of the healthy participants ranged from 35 to 84 years. Thirteen studies enrolled patients with glaucoma of any cause in at least one eye, defined as having both optic disc or RNFL structural abnormalities and visual field defects consistent with structural damage or glaucomatous defects (7, 15–17, 21, 25, 28–33, 37); 11 studies enrolled patients with primary open angle glaucoma (POAG) (13, 14, 18, 22, 24, 26, 27, 32, 34–36); One study enrolled POAG patients as well as PACG patients (20).

For the shape pattern stimulus, 15 studies used full-field illumination paradigms (7, 14, 15, 18, 20–22, 24, 25, 28, 31, 33–35, 37), 10 studies used regional illumination paradigms (7, 15, 20, 22, 24, 25, 27–29, 31), and six studies assessed the visual field defects by functionally-shaped stimuli (e.g., multifocal, Paracentral/Bjerrum/Peripheral) (13, 23, 26, 30, 32, 36). For dark adaptation before stimuli, the dark adaptation time of 11 studies was between 1 and 2 min (14, 15, 17, 18, 20, 21, 25, 28, 31, 33, 34). The other four studies performed 15 s (22, 24), 5 min (30), and 10 min of dark adaptation (35).

Table S3 summarizes a detailed description of CPLRADs. Nine studies employed white stimuli in the standard examination (13, 18, 21–24, 30, 31, 33); 14 studies used colored stimuli (red and/or green and/or yellow and/or blue and/or white) (7, 14–17, 20, 25, 27, 29, 32, 34–37). Sixteen studies assessed the between-eye pupillary response (i.e., the RAPD test) (7, 13, 15, 16, 18, 20–22, 24–26, 28, 29, 31, 32, 36), and 12 studies evaluated the performance of the unilateral PLR test (7, 14, 17, 23, 25, 27, 28, 30, 33–35, 37). Three studies tested both the unilateral and the bilateral matrix (7, 25, 28) (Table 1, Table S2).

### 3.3. Meta-Analysis

**Figure 3:**
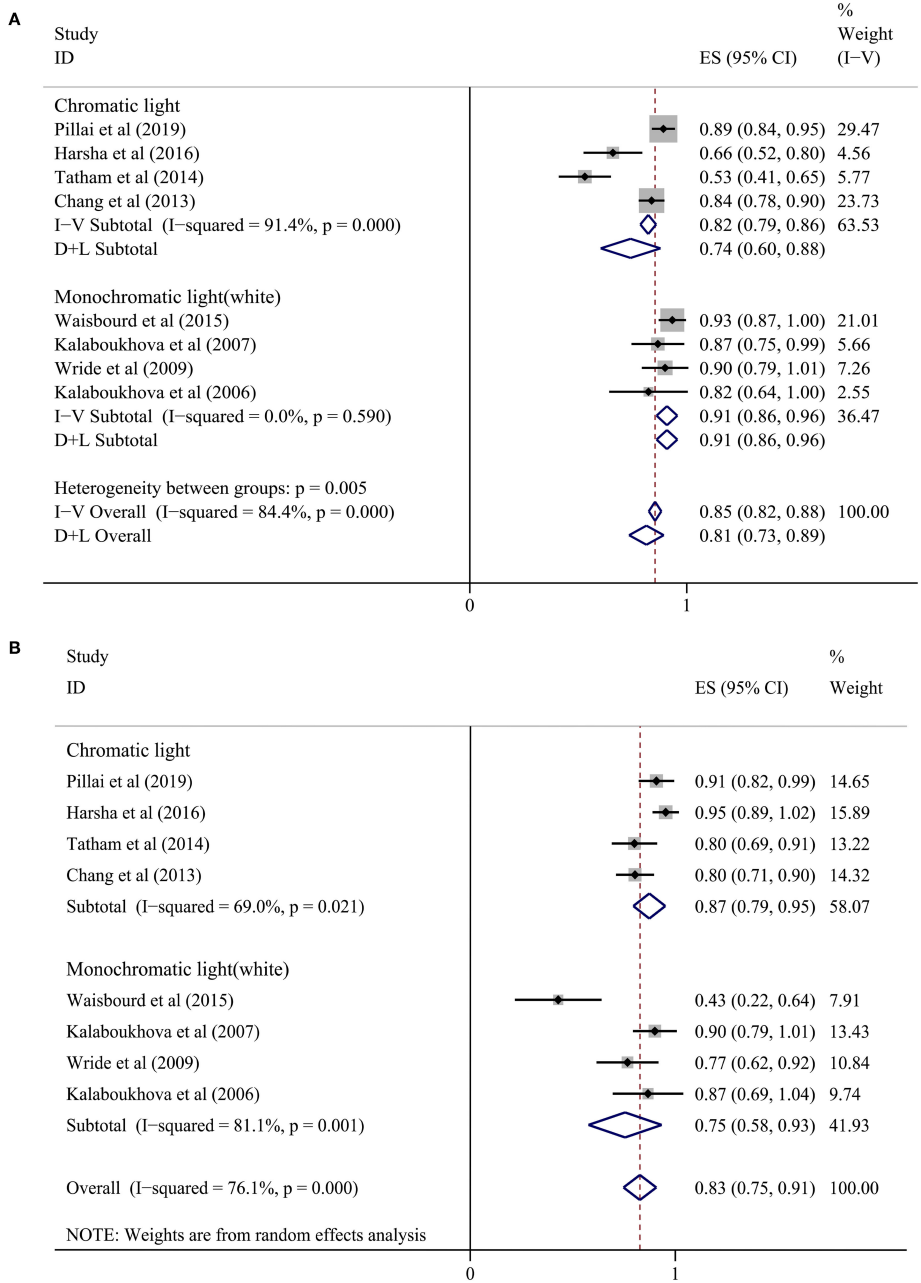
Forest plot showing a summary of sensitivity and sensibility for studies included in the meta-analysis. **(A)** Sensitivity, **(B)** Specificity. A subgroup analysis was conducted between the chromatic light group (CLG) and the monochromatic light (white) group (MLG).

Among our 25 included studies, a control group with normal subjects was not designed in six studies and the accuracy of diagnosing glaucoma by pupillary assessment tests was not stated in the other 11 studies. Thus, our final meta-analysis incorporated eight studies with 829 subjects (Figure 2). A random effect model was chosen within these eight studies because of the significant heterogeneity (*P* < 0.1, *I*^2^> 50%). Figure 3 presents the sensitivity and specificity plot separately and it shows that the estimate of sensitivity was 0.81 (95% confidence interval [CI] 0.73–0.89), and the estimate of specificity was 0.83 (95% CI: 0.75–0.91). The positive likelihood ratio was 4.9 (95% CI: 3.0–8.1), the negative likelihood ratio was 0.21 (95% CI: 0.13–0.33), and the diagnostic odds ratio was 24 (95% CI: 12–48).

In the subgroup analysis, we divided the eight studies into two groups according to the different light stimulus colors (7, 16, 20–25). The sensitivity in the chromatic light group (CLG) (7, 16, 20, 25) was 0.74 (95% CI: 0.60–0.88) and the specificity was 0.87 (95% CI: 0.79–0.95). In the monochromatic light group (MLG) (21–24), the sensitivity was 0.91 (95% CI: 0.86–0.96) and the specificity was 0.75 (95% CI: 0.58–0.93). However, heterogeneity still existed when analyzing sensitivity and specificity in the chromatic group and sensitivity in the monochromatic light (white) group (Figure 3), meaning that there were other causes of heterogeneity.

**Figure 4:**
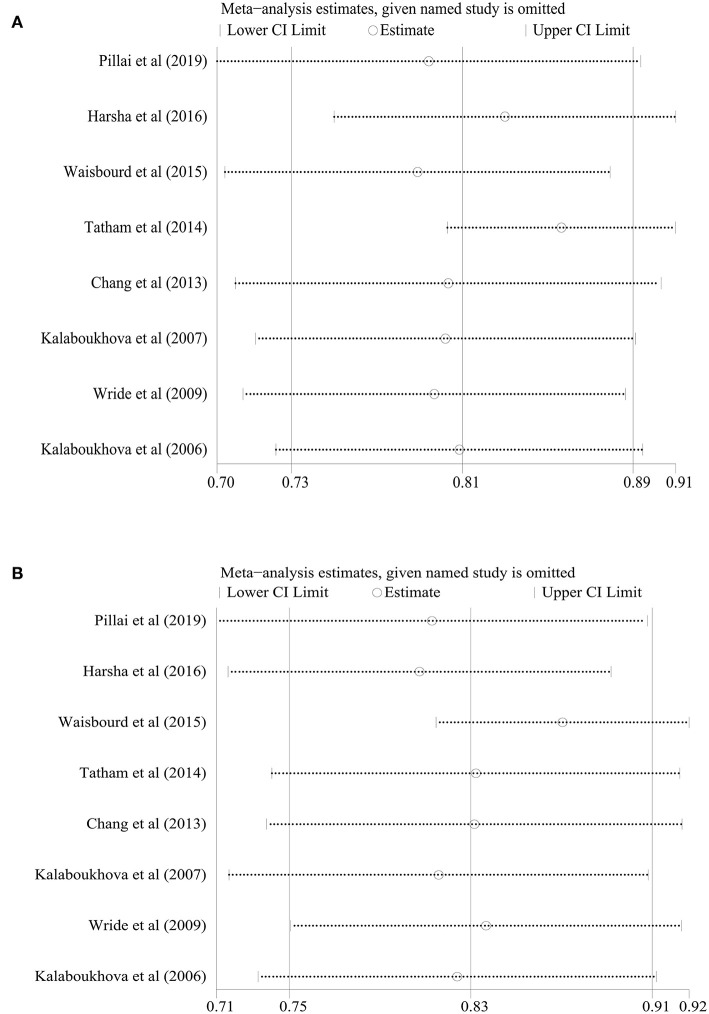
Sensitivity analysis of the included studies. **(A)** Sensitivity, **(B)** Specificity.

We also performed a sensitivity analysis on the synthetic sensitivity and specificity. Figure 4 illustrates that there was obviously no significant change in combined effect values after omitting any one of the studies, which confirmed that our meta-analysis results were stable and reliable.

### 3.4. Quality Assessment

**Figure 5:**
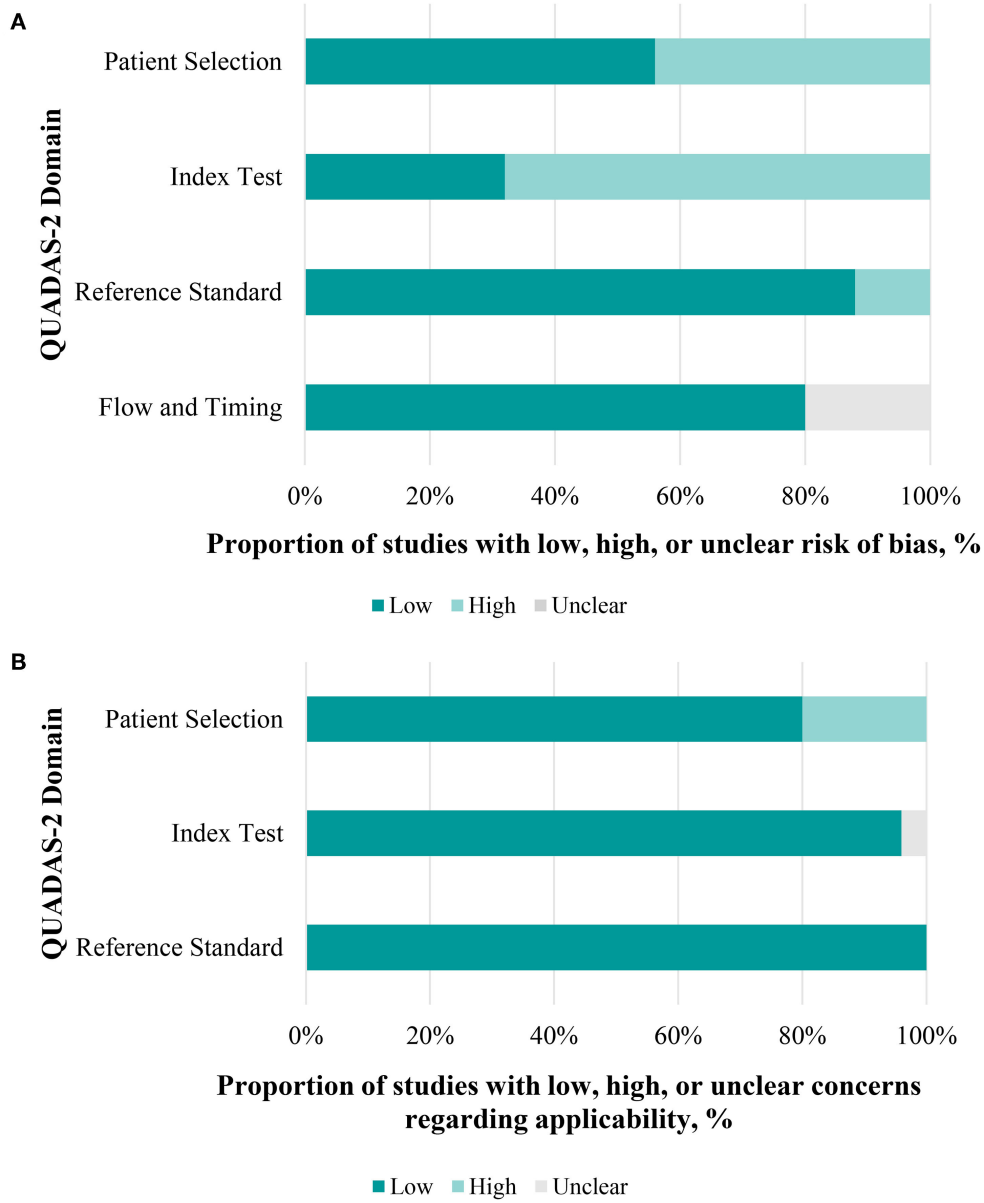
Performance of quality assessment of diagnostic accuracy studies 2 (QUADAS-2) evaluation. **(A)** Each domain in a bar represents the proportion of studies considered low, high, or unclear risk of bias. **(B)** The same applies to concerns of applicability.

Figure 5 provides the overall risk of bias score, and a total of 25 articles that met the inclusion criteria were incorporated in our final analysis after the QUADAS-2 quality assessment. Regarding patient selection, we considered a study to be at high risk of bias when it incorporated patients with unclear types of glaucoma. Studies were considered to be of low applicability concern when non-glaucoma subjects were not designated as a control group. For the index test, we considered studies without statistics on the sensitivity and specificity of diagnosing glaucoma, which may have introduced a high risk of bias. However, there were no major conflicts between the performance or interpretation of the index test and the review question. Concerning the reference standard, most glaucoma patients had been diagnosed by glaucoma specialists according to visual field tests and RNFL thickness. We considered that the patient flow introduced a low risk of bias, because most included studies were conducted in a short period of time, and the delay between the index test (pupillary assessment) and reference standard tests (visual field, RNFL thickness, etc.) would not lead to an effect of the disease progression. A few of studies (15, 16, 20, 21, 33) did not mention the time spent and were thought to be have an unclear risk of bias (Figure 5, Table S4).

## 4. Discussion

Computer-aided PLR assessment devices are drawing increasing attention from both the vision science and the medical equipment engineering domains (4, 38). The present meta-analysis was designed to assess the diagnostic ability of CPLRADs in glaucoma. Overall, CPLRADs introduces objectivity and allow all clinical personnel to conduct the test objectively and reliably in many conditions. Owing to study heterogeneity and variable reference standards, only eight of the 25 included studies qualified for meta-analysis. Our meta-analysis of those eight studies showed reasonably high summary sensitivity and specificity estimates of 0.81 (95% CI 0.73–0.89) and 0.83 (95% CI: 0.75–0.91), respectively (Figure 3). Simpler monochromatic devices, such as Pupilmetrix^TM^ PLR60, generally performed as well as or slightly better than more complex chromatic devices.

The RAPD is recognized as a characteristic finding of glaucoma with various techniques. A previous systematic review and meta-analysis performed by Chang et al. reported data showing that pupillography has a sensitivity of 75% with a specificity of 85% in clinical-based studies by evaluating various methods of RAPD detection including the earlier version of automated pupillography (5). It has also been suggested that older generations of pupillography, including pupil perimetry, infrared video pupillography, and pupil cycle time have higher sensitivity and specificity than the SFM in detecting glaucoma (5). To evaluate the performance of the older generation of automated pupillography with the new version of CPLRADs, we also extracted two studies from this meta-analysis. Our findings are consistent with previous reviews illustrating even more favorable data on the diagnostic accuracy profile of the PLR assessment in glaucoma. Pillai et al. obtained a weighted sensitivity and specificity of 89 and 91.7%, respectively, with an AUC of 0.94 (16).

More importantly, the current state of CPLRADs is more complex than that of the previous earlier version of the automated pupillograph (13, 21, 22). In summary, we consider that the current CPLRADs contain three main parts: an illumination system (infrared illumination, alternating color illumination, background illumination, functionally-shaped stimuli program), an image capturing system (infrared digital camera, monitoring camera), and a computerized image analysis system (capturing the following pupil parameters: baseline pupillary size, maximum contraction velocity, maximum dilation velocity, amplitude (ratio), time to max contraction, time to maximal dilation) (Figure 1, Table S3). In our study, we found that the implementation of monocularly/binocularly monitoring PLR, provides unilateral and bilateral metrics by varying stimulus patterns and colors intensity. With unilateral metrics, 4 of 25 studies compared pupil responses to stimuli between the superonasal and inferonasal fields within each eye (7, 23, 25, 28). Wride et al. found that Pupilmetrix^TM^ PLR60 has a sensitivity of 93.1% with specificity of 76.7% by the peripheral stimulus pattern (Paracentral/Bjerrum/Peripheral) (23). Sixteen of 25 studies used a binocularly monitored automated pupillographic devices, which was designed to record and analyze pupillary responses to various stimuli. The stimuli presented can vary in size, shape, intensity, duration, and color and can be predetermined (7, 13, 15, 16, 18, 20–22, 24–26, 28, 29, 31, 32, 36) (Table 1, Table S2).

Some of the included studies had problems regarding the risk of bias according to the QUADAS-2 quality assessment. For patient selection, participants in all eight meta-analysis studies were enrolled on the basis of different inclusion criteria. Five studies enrolled patients with glaucoma of any cause (7, 16, 21, 23, 25), two of eight studies enrolled POAG (22, 24), and one study enrolled both POAG patients and PACG patients (20). Pillai et al. enrolled patients with glaucoma of any cause (primary angle closure glaucoma post-Yag laser peripheral iridotomy, primary open angle glaucoma, and pseudo exfoliation glaucoma) in at least one eye (16). It is considered that such PACG post-Yag laser peripheral iridotomy would change the PLR matrix to influence the AUC.

The effect of glaucoma severity on the diagnostic ability of CPLRAD parameters and inter-eye asymmetry in MD have a significant effect on the AUCs (7, 20, 21, 25, 33). Rao et al. indicated that the diagnostic performance of automated pupillographic parameters increased when the inter-eye MD difference increased (20). There is evidence of a statistically significant positive coefficient associated with inter-eye MD difference. AUC of (amplitude-full field-white stimulus) Amp-FF-W increased from 0.71 at an inter-eye MD difference of 0 dB (symmetric glaucomatous damage) to 0.93 at an inter-eye MD difference of 15 dB (20). Tatham et al. obtained similar results in their earlier study (7). Waisbourd et al. at a threshold of MD asymmetry >5 dB, pupillography sensitivity was 93.3% (21).

Dark adaptation may be associated with the results of PLR capture to properly evaluate these studies, it is necessary to determine if all the participants underwent proper dark adaptation. In each of the studies, with different periods of time, participants were dark-adapted before light exposure. Eleven of 25 studies allowed 1–2 min for dark adaptation (14, 15, 17, 18, 20, 21, 25, 28, 31, 33, 34). Najjar et al. considered that rhodopsin was not fully regenerated to capture light optimally, and the rods' contribution to the PLR may have been suboptimal (34). Fully regenerating rhodopsin usually requires a dark adaptation of 30–45 min. However, this is time-consuming in the clinical setting. Therefore, future studies could replace this timespan with at least 5 min for partial dark adaptation (~50% of regenerated rhodopsin), especially when using chromatic pupillographs (39).

Simpler monochromatic CPLRADs might be more suitable for practical glaucoma screening. Based on various color stimuli, in our subgroup analysis, the sensitivity in the CLG was 0.74 (95% CI: 0.60–0.88) and the specificity was 0.87 (95% CI: 0.79–0.95). In the MLG, the sensitivity was 0.91 (95% CI: 0.86–0.96) and specificity was 0.75 (95% CI: 0.58–0.93). In addition to considering diagnostic accuracy, the MLG has other advantages over the CLG because the former can be done quickly, is easily reproducible, has a relatively low cost, and avoids more exposure to light radiation.

Using chromatic CPLRADs, however, provides a deeper understanding of rod, cone, and intrinsically-photoreceptive retinal ganglion cells (ipRGCs) contributing to the PLR. The PLR driven by ipRGCs and classical photoreceptors is impaired in glaucoma (14, 17, 27, 32, 34–37). Kankipati et al. indicated that ipRGCs play an important role in the PLR and are primarily responsible for the sustained pupilloconstriciton that occurs after light offset. The amplitude of pupillary constriction is calculated by the percentage of change in the pupillary diameter (PD) between constriction onset and peak constriction in response to each stimulus [(PD resting-PD constricted)/PD resting] (27). Najjar et al. demonstrated that with moderate and high irradiances of blue/red lights, pupillary constriction amplitude is reduced at early stage POAG subjects. This wavelength-independent change demonstrates attenuated ipRGC signaling in the early stages of the glaucoma (34). Kankipati et al. found that participants with glaucoma have a decrease in the ipRGC-mediated post-illumination pupil response (PIPR), which refers to the period of sustained pupillary constriction following light offset (27). Thus, the findings showed that both monochromatic and chromatic CPLRADs have remarkable application prospects in glaucoma detection.

Proper model selection for a larger number of CPLRAD parameters can improve the diagnostic accuracy in glaucoma. Chang et al. demonstrated that using logistic regression including asymmetry in pupillary contraction latency, velocity, amplitude, and age may increase the sensitivity and specificity of pupillography in glaucoma (25). Tatham et al. found that the best AUC was 0.75 with single parameter. However, the best combination of parameters, the AUC was 0.85, which was reduced to 0.74 on cross-validation (7). Above all, to confirm our meta-analysis results, we performed a sensitivity analysis of the synthetic sensitivity and specificity. This illustrates that the results were stable and reliable and that there was no significant change in the combined effect values after omitting any one of the studies (Figure 4).

## 5. Limitation

This study has certain limitations: (1) the search strategy was limited to only those articles written in English; (2) none of our 25 studies, considered how to control the cognitive load and emotional factors that possibly alter both pupil size; and (3) in some of the studies, the glaucoma subjects were notably older than the control subjects. For instance, Rao et al. reported an average age of 61 years for glaucoma patients and 46 years for control subjects (20); (4) Some of the study subjects had systemic conditions, such as diabetes and hypertension and were on medications for these conditions. Moreover, many glaucoma patients were on glaucoma medications with unknown effects on the PLR. Additionally, some other factors may have affected PLR, further limiting the accuracy of the CPLRADs, including the presence of an abnormal pupil shape, previous ocular surgery or topical medications, and systemic; (5) we did not evaluate other computer-aided PLR methods, such as pupil perimetry in glaucoma patients.

## 6. Future Research and Conclusion

A future challenge is to identify the optimum combination of the large numbers of features generated by CPLRADs. As larger sample sizes are available, novel techniques, such as deep learning and image processing can be used to provide better diagnostic ability. It is important to identify that some features perform better on left/right eye stimulation. Furthermore, the application of CPLRADs does not provide any insight into the potential to measure structural damage, such as injury to the optic nerve head (ONH). The development of novel stimuli and assessment may enable calculation of the specific damage to the retina and ONH. If the CPLRADs were to be applied in different settings, such as the community, it might be that their accuracy for glaucoma diagnosis would be lower. Since many factors affect the PLR, further tests are needed to identify the cause of an abnormal PLR.

In conclusion, our results revealed that the diagnostic abilities of even the best CPLRD parameters are only moderate in glaucoma. The diagnostic abilities of the CPLRAD measurements were significantly influenced by the inter-eye asymmetry and within-eye asymmetry in case of glaucomatous damage. Further research on the mechanism of ipRGC in glaucoma should be deeply explored by chromatic pupillography to investigate other factors, such as sleep qualities in glaucoma patients.

## Data Availability Statement

All datasets presented in this study are included in the article/Supplementary Material.

## Author Contributions

LS designed the study and drafted the manuscript. DZ acquired the data and undertook the statistical analysis and interpretation. XQ drafted the manuscript. AL undertook the statistical analysis. YW and CZ revised the manuscript and supervised the study. All authors contributed to the article and approved the submitted version.

## Conflict of Interest

The authors declare that the research was conducted in the absence of any commercial or financial relationships that could be construed as a potential conflict of interest.
