# Supplementary material for: Evaluating State-of-the-Art Computerized Pupillary Assessments for Glaucoma Detection: A Systematic Review and Meta-Analysis
Source: Front Neurol. 2020 Jul 29;11:777. doi: 10.3389/fneur.2020.00777 (PMC7403439; doi:10.3389/fneur.2020.00777)

**Supplementary Material**

# Table S1 Search Strategy

EMBASE

'automated pupil'

'pupillography'/exp OR pupillography 'pupillometer'/exp OR pupilometer 'pupillometry'/exp OR pupillometry

pupillographic OR 'chromatic pupillography' OR (('chromatic' OR chromatic) AND ('pupillography'/exp OR pupillography))

'pupil light reflex' OR (('pupil'/exp OR pupil) AND ('light'/exp OR light) AND ('reflex'/exp OR reflex))

'pupil response' OR (('pupil'/exp OR pupil) AND ('response'/exp OR response)) OR 'pupillary response' OR (pupillary AND ('response'/exp OR response))

'rapd'/exp OR rapd OR 'relative afferent pupillary defect'/exp OR 'relative afferent pupillary defect' OR (('relative'/exp OR relative) AND afferent AND pupillary AND defect) OR 'afferent pupillary defect'/exp OR 'afferent pupillary defect')

('glaucoma'/exp OR glaucoma OR 'ganglion cell'/exp OR 'ganglion cell' OR (('ganglion'/exp OR ganglion) AND ('cell'/exp OR cell))

'glaucoma screening' OR (('glaucoma'/exp OR glaucoma) AND ('screening'/exp OR screening))

'glaucomatous neuropathy'/exp OR 'glaucomatous neuropathy' OR (glaucomatous AND ('neuropathy'/exp OR neuropathy))

'melanopsin'/exp OR melanopsin OR 'intrinsically photosensitive ganglion cells' OR (intrinsically AND photosensitive AND ('ganglion'/exp OR ganglion) AND ('cells'/exp OR cells))

'visual ﬁeld' OR (visual AND ﬁeld) OR 'retinal nerve ﬁber layer' OR (('retinal'/exp OR retinal) AND ('nerve'/exp OR nerve) AND ﬁber AND layer))

Search strategy

('automated pupil' OR 'pupillography'/exp OR pupillography OR 'pupillometer'/exp OR pupilometer OR 'pupillometry'/exp OR pupillometry OR pupillographic OR 'chromatic pupillography' OR (('chromatic' OR chromatic) AND ('pupillography'/exp OR pupillography)) OR 'pupil light reflex' OR (('pupil'/exp OR pupil) AND ('light'/exp OR light) AND ('reflex'/exp OR reflex)) OR 'pupil response' OR (('pupil'/exp OR pupil) AND ('response'/exp OR response)) OR 'pupillary response' OR (pupillary AND ('response'/exp OR response)) OR 'rapd'/exp OR rapd OR 'relative afferent pupillary defect'/exp OR 'relative afferent pupillary defect' OR (('relative'/exp OR relative) AND afferent AND pupillary AND defect) OR 'afferent pupillary defect'/exp OR 'afferent pupillary defect') AND ('glaucoma'/exp OR glaucoma OR 'ganglion cell'/exp OR 'ganglion cell' OR (('ganglion'/exp OR ganglion) AND ('cell'/exp OR cell)) OR 'glaucoma screening' OR (('glaucoma'/exp OR glaucoma) AND ('screening'/exp OR screening)) OR 'glaucomatous neuropathy'/exp OR 'glaucomatous neuropathy' OR (glaucomatous AND ('neuropathy'/exp OR neuropathy)) OR 'melanopsin'/exp OR

melanopsin OR 'intrinsically photosensitive ganglion cells' OR (intrinsically AND photosensitive AND ('ganglion'/exp OR ganglion) AND ('cells'/exp OR cells)) OR 'visual ﬁeld' OR (visual AND ﬁeld) OR 'retinal nerve ﬁber layer' OR (('retinal'/exp OR retinal) AND ('nerve'/exp OR nerve) AND ﬁber AND layer))

PubMed

(automated[All Fields] AND pupil*[All Fields]) OR pupillometer[All Fields] OR pupillograph*[All Fields] OR pupillometry[All Fields]

"pupil light reflex"[All Fields] OR (pupil*[All Fields] AND ("reflex"[MeSH Terms] OR "reflex"[All Fields]))

pupil*[All Fields] AND response* [All Fields]

"pupil disorders"[MeSH Terms] OR ("pupil"[All Fields] AND "disorders"[All Fields]) OR "pupil disorders"[All Fields] OR ("afferent"[All Fields] AND "pupillary"[All Fields] AND "defect"[All Fields]) OR "afferent pupillary defect"[All Fields] OR "RAPD"[All fields]

"glaucoma"[MeSH Terms] OR "glaucoma"[All Fields] OR "glaucomatous" [All Fields] "visual fields"[MeSH Terms] OR ("visual"[All Fields] AND "fields"[All Fields]) OR "visual fields"[All Fields] OR ("visual"[All Fields] AND "field"[All Fields]) OR "visual field"[All Fields]

(("retinal"[All Fields] OR "retina"[MeSH Terms] OR "retina"[All Fields]) AND "nerve fiber layer"[All Fields]) OR RNFL[All Fields]

"ganglion cells"[All Fields] OR "ganglion cell"[All Fields]

"optic nerve diseases"[MeSH Terms] OR ("optic"[All Fields] AND "nerve"[All Fields] AND "diseases"[All Fields]) OR "optic nerve diseases"[All Fields] OR ("optic"[All Fields] AND "neuropathy"[All Fields]) OR "optic neuropathy"[All Fields]

Search strategy

((automated[All Fields] AND pupil*[All Fields]) OR pupillometer[All Fields] OR pupillograph*[All Fields] OR pupillometry[All Fields] OR (chromatic[All Fields] AND pupil*[All Fields]) OR "pupil light reflex"[All Fields] OR (pupil*[All Fields] AND ("reflex"[MeSH Terms] OR "reflex"[All Fields])) OR (pupil*[All Fields] AND response*[All Fields]) OR "pupil disorders"[MeSH Terms] OR ("pupil"[All Fields] AND "disorders"[All Fields]) OR "pupil disorders"[All Fields] OR ("afferent"[All Fields] AND "pupillary"[All Fields] AND "defect"[All Fields]) OR "afferent pupillary defect"[All Fields] OR "RAPD"[All fields]) AND ("glaucoma"[MeSH Terms] OR "glaucoma"[All Fields] OR "glaucomatous" [All Fields] OR "visual fields"[MeSH Terms] OR ("visual"[All Fields] AND "fields"[All Fields]) OR "visual fields"[All Fields] OR ("visual"[All Fields] AND "field"[All Fields]) OR "visual field"[All Fields] OR (("retinal"[All Fields] OR "retina"[MeSH Terms] OR "retina"[All Fields]) AND "nerve fiber layer"[All Fields]) OR RNFL[All Fields] OR "ganglion cells"[All Fields] OR "ganglion cell"[All Fields] OR "optic nerve diseases"[MeSH Terms] OR ("optic"[All Fields] AND "nerve"[All Fields] AND "diseases"[All Fields]) OR "optic

nerve diseases"[All Fields] OR ("optic"[All Fields] AND "neuropathy"[All Fields]) OR "optic neuropathy"[All Fields])

Ovid MEDLINE

(automated AND pupil*) OR pupillometer OR pupillograph* OR pupillometry OR "pupil light reflex" OR (pupil* AND ("reflex" OR "reflex"))

pupil* AND response* OR "pupil disorders" OR ("pupil" AND "disorders") OR "pupil disorders" OR ("afferent" AND "pupillary" AND "defect") OR "afferent pupillary defect" OR "RAPD"

"glaucoma" OR "glaucomatous"

"visual fields" OR ("visual" AND "fields") OR "visual fields" OR ("visual" AND "field") OR "visual field"

(("retinal" OR "retina" OR "retina") AND "nerve fiber layer") OR RNFL "ganglion cells" OR "ganglion cell"

"optic nerve diseases" OR ("optic" AND "nerve" AND "diseases") OR ("optic" AND "neuropathy") OR "optic neuropathy"

Search strategy:

((automated AND pupil*) OR pupillometer OR pupillograph* OR pupillometry OR "pupil light reflex" OR (pupil* AND ("reflex" OR "reflex")) OR pupil* AND response* OR "pupil disorders" OR ("pupil" AND "disorders") OR "pupil disorders" OR ("afferent" AND "pupillary" AND "defect") OR "afferent pupillary defect" OR "RAPD") AND ("glaucoma" OR "glaucomatous" OR "visual fields" OR ("visual" AND "fields") OR "visual fields" OR ("visual" AND "field") OR "visual field" OR (("retinal" OR "retina" OR "retina") AND "nerve fiber layer") OR RNFL OR "ganglion cells" OR "ganglion cell" OR "optic nerve diseases" OR ("optic" AND "nerve" AND "diseases") OR ("optic" AND "neuropathy") OR "optic neuropathy")

# Tables S2 The characteristics of studies not included in the meta-analysis

| Source | Study Design | Age, Y | Device name | B/M | DAT (min) | Stimuli C | Stimuli Pattern | Purpose of study | Main Results |
| --- | --- | --- | --- | --- | --- | --- | --- | --- | --- |
| Pradhan et al, 2017, India | Cross-sectional study  G49, GS10 | G 60±6 | RAPDx | M | 2 min | w | Full field | Predicting the Magnitude of functional and structural damage | Multivariate regression models: Loc and Lmaxc showed the best association with MD (R^2^ of 0.30) |
| Rao et al, 2017a, India | Cross-sectional study  G49, GS10 | G 60±6 | RAPDx | B | 1-2 min | w/b/g/r/y | Central/Peripheral/Full field | Predicting the intereye asymmetry in functional and structural damage | In amplitude change(Ac) percent with white light, best predicted the intereye asymmetry in MD (Intereye asymmetry in MD = 2.20 + 1.33*intereye difference in Ac per cent, R^2^ = 0.36) |
| Sarezky et al, 2016, USA | Cross-sectional study  G34 | G 67.8± 10.9 | RAPDx | B | 2 min | w | Full field | The correlation between PLR defect and inter-eye difference | Standard setting: Correlation coefficients between RAPD score and inter-eye difference in average RNFL thickness, MD, and VFI were -0.81 (P<0.001), -0.73 (P<0.001), and -0.68 (P<0.001). |
| Sarezky et al, 2014, USA | Cross-sectional study G23 | G 67.9± 10.4 | RAPDx | B | 2 min | w | Hemifield/Full field | The correlation between RAPD defect and inter-eye difference | A strong correlation between RAPD scores from full field, bright intensity, white light stimuli and the intereye difference in MD(r=-0.77; P<0.001) |
| Najjar et al, 2018, Singapore | Cross-sectional study  G46, H90 | G63.4±8.3H61.4±8.6 | ETL-100H  Pupillometry | M | 1 min | b/r | Modified Ganzfeld dome | The degree of pupillometric impairment correlates with structural hallmarks of optic nerve damage | Highest correlation coefficients between average RNFL thickness and maximal pupillary constriction obtained at the maximal irradiances of blue(r=0.51, P<0.001), and Red (r=0.45, P=0.002) lights in POAG. |
| Kelbsch et al, 2016, Germany | Case-control study  G25, H16 | G61.8±12.9  H57.8±13.1 | Compact Integrated Pupillograph CiP | M | U | b/r | Full-ﬁeld | Investigate PLR driven by ipRGC and classical photoreceptors in glaucoma | PIPR_blue-red_ was reduced in glaucoma patients compared to normals (p<0.001) and OH (p< 0.01); The extent of VF defects were correlated to SORRS (r = 0.48, p < 0.001), MRAs for red (r = −0.45, p < 0.001) and blue stimulation (r=−0.67, p< 0.001) , PIPR_blue-red_ 11–16 s (r = −0.45, p <0.001) |
| Rukmini et al, 2015, Singapore | Cross-sectional study  G40, H161 | G63.8±6.1  H59.8±6.2 | ETL-100H  Pupillometry | M | 1 min | b/r | Modified Ganzfeld dome | PLRs correlate with glaucoma severity (Impaired ipRGC) | PLR was reduced in POAG only at higher irradiance levels; correlation between pupil diameter and HVF MD(r =-0.44; P = 0.005), and HRT linear C/D ratio (r =0.61; P< 0.001) |
| Martucci et al, 2014, Italy | Retrospective study  G44, H18 | G (1-5):  67.1 ± 7.2  68.5 ± 5.8  69.1 ± 3.2  65.8 ± 4.75  70.8 ± 4.1  H63.2±10.2 | MonCV3 Metrovision | M | 5 min | w | Flashes (unclear) | Evaluate PLR in different stage of glaucoma | Percent pupil contraction (PPC) (p = 0.011), pupil contraction speed (p = 0.001) and minimum diameter (p = 0.019) showed covariate correlation with the stages of glaucoma. |
| Carle et al, 2015, Australia | Case-control study  G19, H24 | G64.1±9.8  H59.8±7.3 | Multifocal Pupillography objective perimetry (mfPOP) | B | U | r/b, b/y | Multifocal | Investigated mfPOP stimuli that target ipRGC in glaucoma | AUC for eyes classified as moderate to severe, blue: 81.7%, yellow: 83.7%  mild disease (AUCs blue: 71.1%, cf. yellow: 67.7% |
| Nissen et al, 2014, Denmark | Case-control study  G11, H11 | G65  H62 | Prototype chromatic pupillometer | M | 1 min | r/b | U | Detect mechanism of ipRGC in unilateral glaucoma | The AUC_0~20s_ to blue light was reduced from 10.80 (unafflicted) to 8.29 (glaucomatous) (p=0.014) and to red light from 8.86 to 6.64 (p=0.035). Both post-illuminatory AUCs (AUC_20–30s_ and AUC_30–50s_) to blue light were significantly reduced. |
| Chang et al, 2013b, USA | Case-control study  G148, H71 | G67±11  H60±10 | RAPDx | B/M | 1 min | U | Peripheral/Full field | Relationship between PLR and Visual field MD and RNFL | The between-eye asymmetry in PLR was significantly associated with visual field MD (R =0.83, P < 0.001), RNFL thickness (R= 0.67, P < 0.001). |
| Duque-Chica et al, 2018, USA | Case-control study  G45, H25 | G65.83±10.2  H54.27±8.98 | an infrared  eye-tracking camera system | M | 10 min | b/r | Ganzfeld | Inner and outer retinal contribution to PLR in glaucoma | Mean RNFL and the PLR SR to the blue flash at 2 log cd/m^2^ (R^2^=0.159; P=0.002) and 2.4 log cd/m^2^ (R^2^=0.200; P<0.001); mean SAP MD and the SR for blue flash at 2 log cd/m^2^ (R^2^=0.090; P=0.018) and at 2.4 log cd/m^2^ (R^2^=0.260; P=0.001) |
| Carle et al, 2011, Australia | Case-control study  G17, H19 | G61.5 ± 9.7  H59.7 ± 8.7 | mfPOP | B | U | b | Multifocal | The diagnostic accuracy of mfPOP in glaucoma | AUROC in VSF protocol: 0.86 ± 0.05 across all visual field severities, (n = 34) and 1.00 ± 0.00 for moderate and severe fields (n = 10). |
| Ozeki et al, 2013, Japan | Case-control study  G58 | G62.6±12.8 | RAPDx | B | U | w/b/g/r/y | Peripheral | Evaluation of RAPD in glaucoma | RAPD amplitudes with differences in MD: white R^2^=0.45 p<0.001, red R^2^=0.18 p=0.002, green R^2^=0.30 p<0.001, blue R^2^=0.29 p<0.001, yellow R^2^=0.32 p<0.001; RAPD latencies with differences in MD: white R^2^=0.17 p=0.001, red R^2^=0.03 p=0.22, green R^2^=0.01 p=0.52, blue R^2^=0.1 p=0.02, yellow R^2^=0.05 p=0.11 |
| Maddess et al, 2009, Australia | Case-control study  G26, H20 | G65.5±10.49  H62.1±6.59 | mfPOP | B | U | w | Multifocal | Assessing visual field defects by recording PLR | The single patch, non-flickered stimulus condition (On1) stimulus produced the best AUC: 84.7 ± 3.9%, |
| Kankipati et al, 2011, USA | Case-control study  G16, H19 | G63.7  H59 | Infrared pupillometry | M | U | b/r | Wide field (60°) | Investigate ipRGC-mediated PIPR in glaucoma | The magnitude of the net PIPR was inversely correlated with the measured visual field loss (mean deviation) of the tested eye (R^2^=0.466, P<0.05) in glaucoma patients. |
| Chen et al, 2008, USA | Case-control study  G40, H40 | G62.2 ±9.0  H52.0 ±5.4 | Computer-based pupillometer | B | U | U | Paracentral/Bjerrum/  Peripheral | Assessing visual field defects by functionally-shaped stimuli | Patient contrast balances were moderately correlated with predictions from perimetric data (r =0.37, p <0.00001); contrast balance and response amplitude combined AUROC was 0.83. |
| Y=year; B/M=Binocular/Monocular monitoring; DAT= Dark adaptation time; Stimuli C= stimuli color; S S Pattern= stimuli shape pattern; U=unclear; w=white; b=blue; g=green; r=red; y= yellow; AUROC=area under the receiver operating characteristics curve; G=glaucoma patients; H=health control; OH=ocular hypertension; GS=glaucoma suspect; asy=asymmetry; MD=Mean Divation; CDR=cup to disc ratio; mfPOP=Multifocal Pupillography objective perimetry; PIPR= post-illumination pupil response; U=unclear. Loc=Latency of onset of constriction; Lmaxc=Latency of maximum constriction; RNFL= retinal nerve fibre layer; VFI=visual field index; ipRGC=intrinsically photosensitive retinal ganglion cells; SORRS=slope of the response during exposure to the 4 s red stimulus; MRA=Maximal relative amplitude; HVF=Humphrey Visual Field; HRT=Heidelberg Retinal Tomography; SR=sustained response; SAP=standard automatic perimetry) VSF=very sparse flicker | | | | | | | | | |

# Tables S3 Characteristic of computerized pupil assessment devices

| Device Name | Enrolled Study | Device Description |
| --- | --- | --- |
| RAPDx | Rao et al., 2017a, Pradhan et al. 2017, Rao et al., 2017b, Sarezky et al., 2014, Waisbourd et al., 2015, Tatham et al., 2014, Sarezky et al., 2016, Chang et al., 2013a, Chang et al., 2013b, Ozeki et al., 2013 | RAPDx (Expanded pupil diagnostics, Konan Medical, Irvine, CA, USA) is a commercially available computerized automated pupillography device. Pupil videography was performed using a pair of 60 Hz infrared cameras mounted at 35-degree angles with a peak emission of 880 nm. The cameras had a resolution of 240×240 pixels/frame for approximately 25 pixels/mm, and any blinks that occurred during the critical portions of the pupil response resulted in deletion of that stimulus-response pair from analysis and automatic repeat testing. The machine synchronously recorded spatial (pupillary diameter) and temporal (latency) data. The response of both pupils is recorded using infrared cameras and a graph is generated displaying pupil diameter (PD) against time. |
| RAPiDo | Pillai et al., 2019 | Binocular system RAPiDo (Neuroptics Inc., Irvine, CA, USA) is the direct consequence of a servo-analytic modeling to the pupil. It is a portable, battery-operated binocular pupilometer. |
| A custom‐built pupillometer | Kalaboukhova et al., 2006, Kalaboukhova et al., 2007 | A custom‐buil pupilometer consist with two separate video cameras; infrared diode lamp for background illumination; two white diode lamps generating light stimuli; two pasteboard screens. |
| Pupilmetrix™ PLR60 | Wride et al., 2009 | Pupilmetrix™ PLR60 (Applied Neurodiagnostics Ltd, Cramlington, UK) It combines the established and well  understood PLR with infrared pupilometry (IRP) technology to provide data on the response of the pupil to a series of specially designed stimuli. Using specially designed software the device calculates a result that indicates the presence or absence of asymmetric damage to the nerve fiber layer of the retina. |
| Compact Integrated Pupillograph CiP | Kelbsch et al., 2016 | One eye was stimulated under mesopic conditions with 28 lx bright light, either red (605 nm± 20 nm) or blue (420 nm ±20 nm), provided by a mini-Ganzfeld color LED stimulator (CH Electronics). The consensual pupil light reaction of the fellow eye was recorded by means of infrared pupillometry (Compact Integrated Pupillograph CiP by AMTech Germany) over a period of 16 s including a prestimulus time of 5 s. |
| ETL-100H  Pupillometry | Najjar et al., 2018, Rukmini et al., 2015 | To measure the direct PLR, light was administered to one  eye using a modified Ganzfeld dome (Labsphere, Inc, North Sutton, NH), with the other eye covered by a patch. Subjects were exposed to a blue-light stimulus (469 nm) or red-light stimulus (631 nm), with the order of exposure randomized and counterbalanced. Narrow-bandwidth light was provided using lightemitting diodes (Nichia Corporation, Tokushima, Japan) that were controlled using a function generator (Keithley Instruments, Inc, Cleveland, OH). |
| MonCV3 Metrovision | Martucci et al., 2014 | Monocular dynamic pupillometry (MonCV3 Metrovision)  must have been performed in both eyes (one eye at a time by occluding one eye) in darkness after 5 min of darkness adaptation, for a duration of 90 s. Patients must have been examined using white light flashes (stimulation ON time 200 ms, stimulation OFF time 3,300 ms, total luminance 100 cd/m^2^, total intensity 20 cd.s/m^2^). The stimulator is equipped with near-infrared illumination (880 nm) and a high-resolution near infrared image sensor which allows measurement of pupil diameter even in complete darkness. The images of the eyes are acquired and processed in real time (30 images per second). |
| Multifocal Pupillography objective perimetry (mfPOP) | Carle et al., 2011, Carle et al., 2015, Maddess et al | Presentation of mfPOP stimuli and monitoring of pupil diameter were carried out using a prototype of the US Food and Drug Administration–approved nuCoria Field Analyzer (nuCoria Pty. Ltd., Acton, Australia). This tabletop device uses concurrent, dichoptic presentation of temporally and spatially sparse multifocal stimuli at 60 frames/s. Infrared light was used to illuminate subjects’ pupils and their responses were monitored by separate video cameras at 30 frames/s/eye. |
| Prototype chromatic pupillometer | Nissen et al., 2014 | The instrument consists of two parts: an input section, which stimulates one eye for a predetermined time period(usually20s) with light of a well-defined wavelength and luminance, and an output section, detecting the area of the contralateral pupil before, during, and after light stimulation. Both sections are controlled by a common computer program and thus synchronized. The area of the pupil is monitored with a frequency of 20Hz and converted into a diameter, assuming a circular pupil. Light intensity (luminance) was 300cd/m^2^ for red and blue light, corresponding to 10 quanta/cm2/s (red) and 10 quanta/cm2/s(blue) and less for the infrared detecting system. All intensities were chosen well below the recommendations of ANSI-2007 and ICNIRP. Initial calibration was performed with the RP-655 spectrophotometer (Photo Research, Chatsworth, CA, USA). |
| An infrared eye-tracking camera system | Duque-Chica et al., 2018 | Stimuli consisted of a 1s blue (470 nm) and red (640 nm)  light flashes, generated by a Ganzfeld (Q450, Roland), controlled by the A-pattern simulation system (RETI-port; Roland Consult, Brandenburg, Germany). They were presented with the following photopic range of luminances: −3, −2, −1, 0, 1, 2, 2.4 log cd/m^2^. The Ganzfeld was factory calibrated, we have verified this with a Konica Minolta CS-100A luminance and color meter for the luminance levels (0, 1, 2, 2.4 log cd/m2). For luminance levels below these, we used extrapolation of the measured values. The red and blue stimulations were created with the specific light-emitting diodes of the Ganzfeld, the light emitting diodes had peak wavelengths of 470 nm (blue) and 640 nm (red) and Full width at half maximum of 26 nm and 17 nm, respectively. |
| Infrared pupillometry | Kankipati et al., 2011 | A novel optical system that has previously been described was used for this study. The viewing eye of the subject was dilated with 1% tropicamide (Mydral; Bausch & Lomb, Rochester, NY) and 2.5% phenylephrine (Neofrin; Bausch & Lomb), and the light stimulus was presented to this eye while the consensual pupil response in the undilated eye was recorded by infrared camera (Digivue EC-PC-Cam; Elyssa Corporation, Briarcliff Manor, NY) and computer. |
| Computer-based pupillometer | Chen et al., 2008 | Stimuli were presented on a cathode ray display controlled by a computer. Horizontal pupil diameter was recorded (60 samples/sec) using a computer based pupillometer. The three stimulus pairs were named “paracentral” (the smallest stimulus pair, nearest to fixation and near the horizontal meridian between fixation and the blind spot), “Bjerrum” (a larger stimulus pair, further from fixation and covering much of the Bjerrum area), and “peripheral” (furthest from fixation and extending 20 deg into the nasal field). |

**Tables S4 Quality Assessment using QUADAS-2 Tool.**

We used the QUADAS-2 Tool to examine the study quality, which covered the risk of bias and applicability concerns of patient selection, index test, reference standard, and flow and timing.


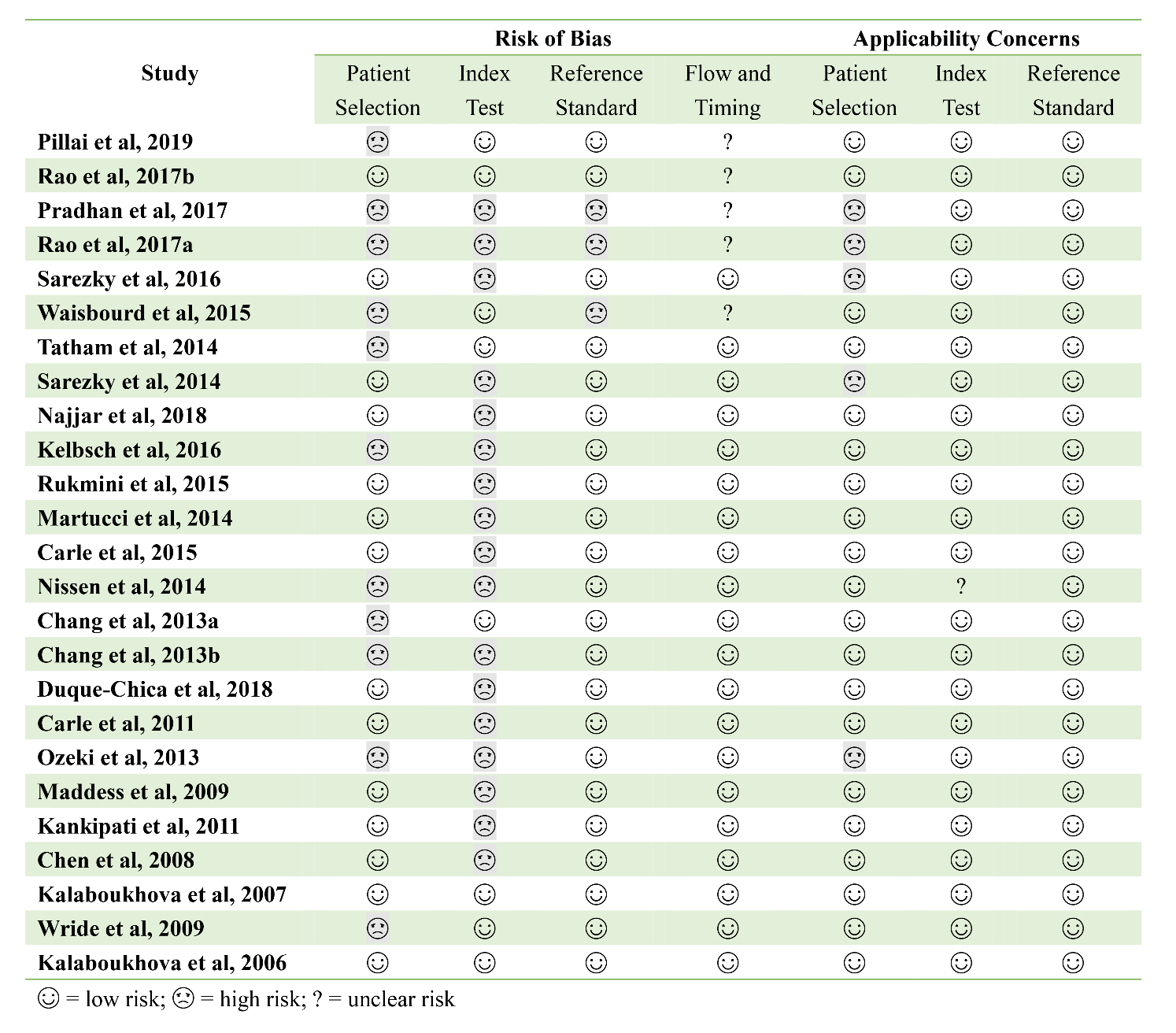

Supplement: Supplementary file 1 [file Data_Sheet_1.docx]
